# Refractory Head and Neck Lymphatic Malformation in Infants Treated With Sirolimus: A Case Series

**DOI:** 10.3389/fonc.2021.616702

**Published:** 2021-07-16

**Authors:** Changhua Wu, Dan Song, Lei Guo, Liang Wang

**Affiliations:** Department of Vascular Anomalies and Interventional Radiology, Qilu Children’s Hospital of Shandong University, Jinan, China

**Keywords:** lymphatic malformation, sirolimus, head and neck, sclerotherapy, infant

## Abstract

**Background:**

Extensive and complex head and neck lymphatic malformations (LMs) are challenging to manage through traditional therapy. The purpose of this retrospective study was to assess the efficacy and safety of sirolimus in infants with refractory head and neck LMs.

**Methods:**

Sirolimus was administered orally on a continuous dosing schedule. Patients were seen every month for the first three months and then subsequently every three months. The primary endpoints were safety and efficacy based on clinical and radiological evaluations.

**Results:**

Eight patients, refractory to standard care, were enrolled and received sirolimus continuously. After 12 months of follow-up, the response and safety to medication was evaluated: all patients experienced reductions in LMs bulk, ranging from modest to significant. Some minor adverse effects were reported: mouth sores, eczema, gastrointestinal reaction, dyslipidemia, and neutropenia.

**Conclusion:**

Sirolimus was efficient in children with refractory head and neck LMs and was well tolerated.

## Highlights

1. The purpose of this retrospective study was to assess the efficacy and safety of sirolimus in infants with refractory head and neck lymphatic malformation.
2. Extensive and complex head and neck lymphatic malformation are challenging to manage through traditional therapy such as sclerotherapy and surgery. Sirolimus has been utilized to treat a host of vascular anomalies. While the efficacy and safety is unclear now in infants.
3. This article will be the complement to existing researches.

## Introduction

Lymphatic malformations (LMs) are low-flow vascular anomalies of the lymphatic system that arise from PIK3CA mutations in lymphatic endothelial cells (1, 2). The neonatal incidences of LMs are estimated to be 1.2-2.8 out of 1000 births (3), and most frequently affect the head and neck (4). They can be characterized into common (cystic) LMs (including three subtypes: macrocystic, microcystic and mixed cystic), generalized lymphatic anomalies (GLA), LMs in Gorham-Stout disease, channel-type LMs, primary lymphedema and others according to the International Society for the Study of Vascular Anomalies (ISSVA) classification (5). A further staging system for head and neck LMs (HNLM) has been suggested based on the anatomic location and laterality of lesion (6, 7).

LMs in the head and neck may result in significant mortality due to airway constriction secondary to mass constriction from an adjacent macrocyst, laryngeal or bronchial microcystic LMs, or circumferential subglottic LMs. In addition, LMs in this location may impair sound and eating due to their size or secondary infection (8–10). Thus, the therapeutic goals must to first address functional impairment, particularly those that are life-threatening, and secondly improve esthetic impairment and pain. Surgical resection as the primary form of treatment may allow a sharply defined cystic lesion for complete curative excision with a single procedure such as macrocysticlymphatic malformations. However, surgery for infiltrative and/or extensive lesions such as microcystic LMs remains challenging becaused of the risk of cosmetic deformity, fistula formation, vascular damage, and cranial nerve injury (9, 11). Sclerotherapy of macrocysticlymphatic LMs, particularly using bleomycin (12) or OK-432 (13) has achieved good clinical efficacy. However, the great challenge in sclerotherapy of large microcystic and mixed LMs, which rarely are surgically excisable given the infiltrative pattern, is limited regression. This procedure, while not surgical resection, does not generally carry possible complications such as vascular damage, and cranial nerve injury However, the characteristic complication of sclerotherapy, such as the buildup of scar tissue and growing swelling, is unavoidable (14). Airway stenosis caused by swelling of LMs is a life-threatening complication especially in the head and neck (14). In our experience, individualized sequential multi-therapy is the key factor in reaping huge benefits, which include sclerotherapy for cystic areas and medical therapy followed by plastic surgery.

Sirolimus, a mammalian target of rapamycin (mTOR) inhibitor, has been utilized to treat a host of vascular anomalies despite that was not approved by Drug Instructions (15, 16). This plays a key role in inhibiting angiogenesis and cell growth through greater regulation of the mTOR pathway. Six patients with complicated vascular anomalies including Kaposiform hemangioendothelioma with Kasabach–Merritt phenomenon (KMP), diffuse microcystic LMs, and capillary lymphatico-venous malformation were reported to respond to sirolimus with tolerable side effects (17). Recently, sirolimus was evaluated in a phase 2 study in 19 patients with extensive and/or complex slow-flow vascular malformations, with significant and rapid improvement of their symptoms and quality of life (18). We performed a retrospective study to evaluate the efficacy and safety of sirolimus in infants with extensive and/or complex LMs refractory to standard treatments.

## Methods

### Patients

Eight patients, refractory to standard care, were enrolled and received sirolimus continuously. Inclusion criteria included symptomatic head and neck extensive microcystic or mixed LMs that were refractory to standard care, such as sclerotherapy and/or resection procedures. Respiratory tract obstruction, bleeding and/or coagulation abnormality were the main symptoms considered for inclusion in the study. There were no age limitations. The eligible patients had to have adequate liver (bilirubin, ASAT, ALAT), medullar (neutrophils≥1500/mm^3^, hemoglobin≥8.0 mg/dL and platelets≥0.000/mm^3^) and renal (clearance≥70 ml/min/1.73m^2^) function with a Karnofsky performance status≥50. If pediatric tracheotomy is performed, all responses can be attributed to sirolimus.

Exclusion criteria included severe, concurrent and/or uncontrolled diseases (cardiopathy, diabetes, infection, human immunodeficiency virus, hypertension…), concomitant CYP3A4 inhibitor/inducer intake, and gastro-intestinal disorders that may modify sirolimus absorption. Patients could not undergo surgical resection and/or sclerotherapy within four weeks prior to study entry. Exclusion criteria also included previous use of an mTOR inhibitor.

### Study Protocol and Treatment

Careful and complete examination before sirolimus is important for treatment such as complete blood count, blood chemistries, D-dimer and fibrinogen level measurements. Magnetic resonance imaging (MRI) studies included T1- and T2-weighted imaging with fat saturation and/or STIR sequences in two orthogonal planes are necessary.

Sirolimus was started at a dose of 0.8 mg/m^2^ body surface area, twice-a-day, using liquid solutions for all patients. All patients were seen every two weeks to measure the sirolimus serum level and the doses were adjusted until two successive blood levels (10-15ng/mL) were obtained. Then, all patients were seen months to evaluate their compliance, the signs and symptoms of the malformation, and possible side effects of the drug. Hemogram, liver and renal function, and coagulation parameters were monitored monthly when the blood level reached a plateau. If the patient experienced hepatotoxicity, the sirolimus dose was decreased.

The primary endpoint of the treatment was complete response or functional remission, and no evidence of recurrence six months after therapy discontinuation. Treatment was suspended in cases of intolerance toxicity and/or no responses can be attributed to sirolimus after three months of therapy.

The primary endpoint of this study was the efficacy and safety of sirolimus after the third, sixth, and twelfth months. The response to medication was evaluated based on reduction in LM bulk by clinical examination, photographs, and radiologic imaging when available. A global self-evaluation percentage (0% = no change to 100% = symptom free; improvement was considered as weak between 0 - 20%, moderate between 20 - 50% and strong when ≥ 50%) was recorded for each patient and/or parent at each consultation.

### Ethics Committee Approval

The protocol was approved by the institutional review board of Qilu Children’s Hospital of Shandong University.

## Results

Eight patients [5 females and 3 males; from 2months to 3 years old (median: 11.89 ± 13.11 months)] were enrolled between January 2016 and January 2018. Two patients had microcystic LM and six had mixed macrocystic-microcystic LMs (**Table 1**). All patients had previously been treated with several sequences of sclerotherapy and/or surgical resection. All had severe symptoms varying from esthetic deformation (n = 8), respiratory tract obstruction (n=6), sucking dysfunction (n=4), bleeding (n=5), and/or anemia (n=3). All patients received sirolimus for at least 12 months; one restarted after a break of around three weeks because of pneumonia. All patients were still on treatment, with a follow-up ranging from 12 - 18 months.

**Table 1:** Patient Characteristics and Sirolimus Response.

Patients	LM Involvement	LM Type	Age at Initiation	Sirolimus Duration(years)	Response				Sirolimus Toxicities	Time off Sirolimus	Previous treatment
					Reduction	Mucosal Vesicles	Airway	Rate of Infection/Bleeding			
1	Extensive bilateral cervicofacial with tongue, parotid gland, floor of mouth, oxygen-dependent	Mixed, mostly Microcystic	2.5 months	1.33	Significant	Improved	Began trachea cannula then spontaneously breathing	Decreased bleeding	Mouth sores	NA	Sclerotherapy
2	Extensive bilateral cervicofacial with submentum, Anterior chest wall	Mixed	2.4 months	1.38	Significant	NA	Began trachea cannula then spontaneously breathing	Decreased bleeding	Mouth sores, neutropenia	NA	Sclerotherapy
3	Extensive right cervicofacial, left hemilarynx, posterior pharynx, supraclavicular region	Mixed	9.25 months	1.11	Modest	Improved	Began trachea cannula then spontaneously breathing	Decreased bleeding	Mouth sores, repeated infection	Drug withdrawal due to pneumonia for one month	Sclerotherapy
4	Extensive bilateral cervicofacial, Buccal mucosa, anterior and base of tongue, Right nasopharynx and oropharynx, soft palate	Mixed, mostly macrocystic	2 months	1.29	Significant	Improved	Began oxygen-dependent then improvement of sleep quality	Decreased bleeding	Mouth sores, eczema	NA	Sclerotherapy
5	Extensive bilateral cervicofacial	Mixed, mostly Microcystic	2 years, 4 months	1.65	Modest	Improved	Improvement of sleep quality	NA	Emesis, Mouth sores	NA	Sclerotherapy
6	Right cervicofacial, floor of mouth, mediastinum superior	Mixed, mostly Microcystic	1 year	1.52	Modest	Improved	NA	NA	Diarrhea, elevated cholesterol and triglycerides	NA	Surgical resection
7	Maxillofacial region, submandibular	Microcystic	3 years	1.23	Modest	Mild improved	NA	Bleeding episodes improved	NA	NA	Sclerotherapy
8	Left cervicofacial	Microcystic	3 months	1.36	Modest	NA	Improvement of sleep quality	Decreased bleeding	Mouth sores	NA	Sclerotherapy

**Figure 1:**
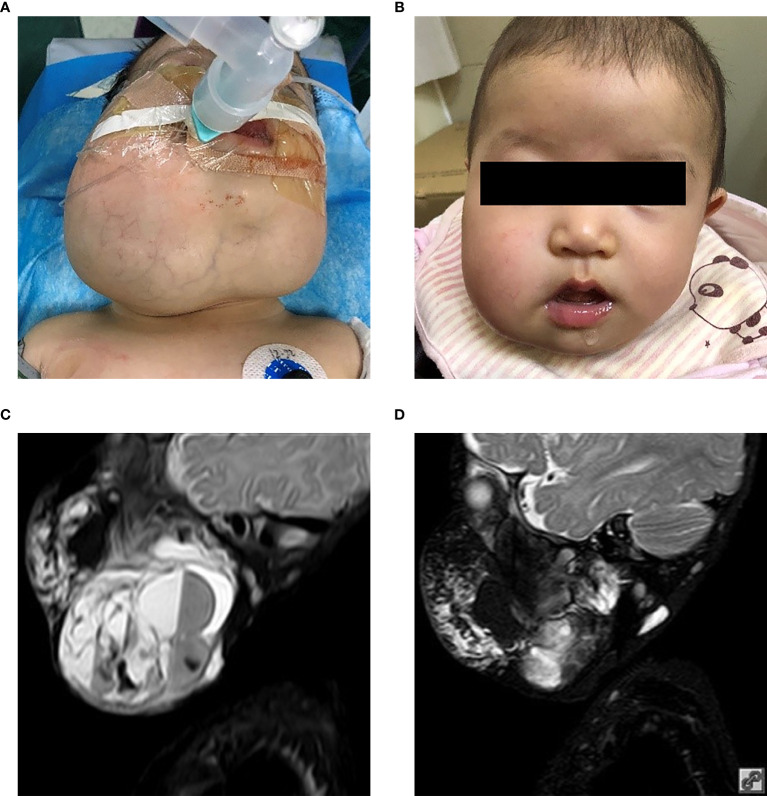
Patient 2 from **Table 1**. Extensive mixed, mostly macrocystic cervicofacial lymphatic malformations requiring intubation at birth. **(A)** Baseline after sclerotherapy, prior to initiation of sirolimus. **(B)** Five months on sirolimus. Sirolimus initiated at 5 months of age. T2 magnetic resonance imaging upon **(C)** initiation of sirolimus and **(D)** response after 5 months. T2, transverse relaxation.

In patients with visible LMs sirolimus resulted in a noticeable improvement in appearance compared to previous photographs in all patients at the one year time-point. An example of a significant sirolimus response in a young child with significant cervicofacial LMs is shown in **Figure 1**. This improvement appeared within three months from the start of sirolimus in all patients and persisted at the 6- and 12-month evaluation points. In addition, appearance improvement was more significant in the first three months. Similar to the oral mucosal response, the airway mucosa also responds well to sirolimus. In this study, six patients showed with mucosal vesicles, which were significantly reduced in five patients and mildly improved in one patient (#7).

**Figure 2:**
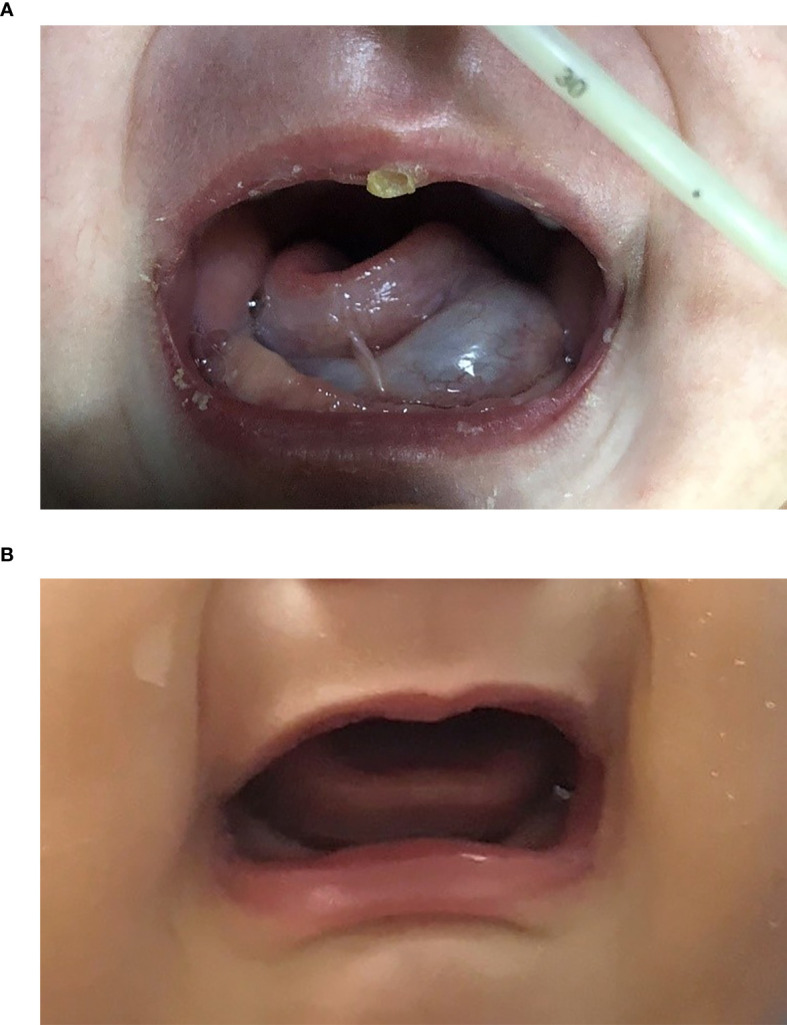
Two-month-old female (patient 4 in **Table 1**) with extensive bilateral microcystic cervicofacial lymphatic malformations with tongue and floor of mouth involvement at **(A)** start of therapy, and **(B)** at 6 months. This patient also has extensive bilateral submandibular and facial involvement, which also significant regression during sirolimus therapy (not shown).

The improvement in respiratory function and sucking were observed in all patients according to clinical symptoms. These appeared within one month from the start of sirolimus and were maintained. One patient had respiratory tract obstruction with mixed LM after numerous sclerotherapy procedures; the obstructive dyspnea improved with regression of the lesion in the first month without tracheostomy (**Figure 2**). Intubation was required initially for four patients.

Intracystic hemorrhage was a common cause of respiratory tract obstruction in four patients(#1,#2,#3, and #4) and a mild narrowing of the upper airway in one patient(#8). Two patients showed repeated bleeding during sclerotherapy (#1, and #3), which stopped completely until sirolimus.

Among the eight patients, radiological evaluation is difficult through quantitative evaluation at 12 months of treatment. The lesions showed a very infiltrative and/or multifocal sign on T2-weighted imaging, which caused an inadequate and unreliable contouring of the lesions. Visual evaluation of the improvement of LMs was performed. T2-MRI-weighted sequences showed a size reduction in seven of them (87.5%) at three months: 30% reduction in lesion size in five patients, and 40% in two patients. Despite clinical improvement, the absence of a response to malformation was observed in one patient (#7). At six months, a reduction in lesion size by 40% was observed in six patients, 60% in one patient, and 10% in one patient. At the final observation (12 months): a reduction in lesion size by 40% was observed in three patients, 60% in three patients, 75% in one patient, 10% in one patient.

Sirolimus was effective and well tolerated in infants. Some minor adverse effects were reported: mouth sores (6/8), eczema (1/8), gastrointestinal reaction (2/8), dyslipidemia (1/8), and neutropenia (1/8) (NCI Grade 2). Only one patient interrupted sirolimus therapy for recurrent fever caused by upper respiratory tract infection (#3) (NCI Grade 3).

## Discussion

This study found that sirolimus was effective for refractory head and neck LM in infants, with acceptable complication rates. Our experience suggests that the macrocystic part may respond better than the microcystic part in the same patient, which may be due to reduced cyst fluid production by further research is needed. As respiratory tract obstructions caused by intracystic hemorrhage may be promptly reversed through percutaneous aspiration of fluid in the cyst and injection of bleomycin, the authors advocate for early use of sirolimus to avoid tracheostomy. Mucosal disease, particularly involving the lingual resion, consistently responds to sirolimus earlier than neck lesions. Our results also suggest that younger patients may respond better than older patients.

Sirolimus revolutionized the treatment of LMs and combined lesions, targeting the rapamycin (mTOR) pathway, resulting in rapid involution of vascular anomalies (19). In 2011, Hammill et al. retrospectively evaluated a series of four infant patients with diffuse microcystic LMs (17). All patients showed significant improvement in clinical status with tolerable side effects. A monocentric prospective phase II study also confirmed the reported data (18). Recent studies have shown that neonates born with complex LMs can also benefit from sirolimus therapy despite these models being limited in sample size (20–22). However, a proposed dosing regimen for very young infants may require further study. Several studies have modeled the therapeutic drug management of sirolimus, which covered cohorts of different ages (21, 23). The current literature supports the efficacy of sirolimus for the treatment of LMs. However, a report on sirolimus completely curing patients with LMs has not been reported. And the optimal timing for ending sirolimus treatment is still an important and unsolved problem.

Four patients with cervicofacial LMs were described to have exacerbations and challenged airway patency in our study. All of them accepted airway management, such as trachea cannula or oxygen dependency and achieved remission in a short time through sclerotherapy and sirolimus. This led the authors to support the short-term efficacy of sirolimus in LMs and advocate for early use of sirolimus to avoid tracheostomy. Laforgia et al. reported that a case of LMs with respiratory distress in a newborn was significantly reduced after only five days of sirolimus therapy (20). This impressive rapidity of action highlights the efficacy of sirolimus in life-threatening LMs.

It has been verified that various complex vascular anomalies including LMs can also benefit from sirolimus (17, 18, 24, 25). Notably, no serious toxicities were reported while receiving sirolimus therapy. Sirolimus was associated with mouth sores, hypercholesterolemia, headaches and neutropenia, which could be successfully managed by symptomatic or low-dose treatment. Many authors recommend that patients should receive Pneumocystis prophylaxis with trimethoprim-sulfamethoxazole, yet the research about this is inadequate. Infection tended to be milder and less frequent after treatment with sirolimus (26), which is consistent with this study. In our study only a young patient presented with repeated infection after one month of treatment in our study. While it is hypothesized that the immonusuppressive effect of sirolimus resulted in repeated infection, we could not completely exclude other causes because this patient was treated with sirolimus only for a short period of time. Although serious complications have not been reported, follow-up of patients treated with an mTOR inhibitor is necessary and should include imaging (protopathy an bone marrow) and hematological controls (hemogram, liver function, and immune cells) every two months.

Our study is limited by the number of patients enrolled due to the rarity of refractory head and neck LMs in infants. Second, the heterogeneity of the malformations as an influencing factor also hindered the establishment of an efficacy evaluation criterion. The use of multimodal treatment may be another influencing factor because it may confound the effect size attributed to sirolimus therapy. All enrolled infants underwent surgical resection or sclerotherapy before sirolimus. Lastly, there is a lack of long-term follow-up, so the long-term toxicities of sirolimus remain unknown.

Hence, the main benefit of this study is the resolution of uncertainty concerning the efficacy and safety of sirolimus in children with refractory head and neck LMs. In addition, we demonstrated the early response of sirolimus in life-threatening LMs. Of note, no serious toxicities were reported in the short-term follow-up of sirolimus therapy.

## Data Availability Statement

All datasets presented in this study are included in the article/supplementary material.

## Ethics Statement

The studies involving human participants were reviewed and approved by The institutional review board of Qilu Children’s Hospital of Shandong University. Written informed consent to participate in this study was provided by the participants’ legal guardian/next of kin for the publication of any potentially identifiable images or data included in this article.

## Author Contributions

CW: Drafting the article, substantial contributions to conception and design, final approval of the version to be published. DS: Acquisition of data, or analysis and interpretation of data. LG: Final approval of the version to be published. LW: Acquisition of data, or analysis and interpretation of data. All authors contributed to the article and approved the submitted version.

## Conflict of Interest

The authors declare that the research was conducted in the absence of any commercial or financial relationships that could be construed as a potential conflict of interest.
